# DNA-Mediated Interferon Signature Induction by SLE Serum Occurs in Monocytes Through Two Pathways: A Mechanism to Inhibit Both Pathways

**DOI:** 10.3389/fimmu.2018.02824

**Published:** 2018-12-11

**Authors:** Amit Porat, Eitan Giat, Czeslawa Kowal, Mingzhu He, Myoungsun Son, Eicke Latz, Ilan Ben-Zvi, Yousef Al-Abed, Betty Diamond

**Affiliations:** ^1^Elmezzi Graduate School for Molecular Medicine, Manhasset, NY, United States; ^2^Center for Autoimmune Musculoskeletal and Hematopoietic Diseases, Feinstein Institute for Medical Research, Manhasset, NY, United States; ^3^Center for Molecular Innovation, Feinstein Institute for Medical Research, Manhasset, NY, United States; ^4^Biomedical Centre (BMZ), Institute of Innate Immunity, 1G007 University Hospital, University of Bonn, Bonn, Germany

**Keywords:** type 1 interferon, receptor for advanced glycation end products (RAGE), Fc receptor gamma 2a, monocytes, systemic lupus erythematosus (SLE)

## Abstract

A primary mechanism for activation of innate immunity is recognition of damage or pathogen associated molecular patterns by pattern recognition receptors (PRRs). Nucleic acid is a damage associated molecular pattern molecule that when internalized into a monocyte and recognized by intracellular nucleic acid sensing toll like receptors will cause production of type 1 interferon. The process by which DNA or RNA is delivered into the cytosol of monocytes in systemic lupus erythematosus remains incompletely understood, and therapeutic approaches to prevent DNA-mediated monocyte activation are needed. We identified two mechanisms for internalization of DNA by monocytes. IgG-bound DNA was internalized by interacting with Fc gamma receptor IIa, while high-mobility group box-1 protein-bound DNA was internalized by interacting with the receptor for advanced glycation end products. Both pathways contribute to an inflammatory phenotype in monocytes exposed to serum from patients with SLE. Moreover, both of these pathways can be inhibited by a pentapeptide, DWEYS, which is a DNA mimetope. In one instance DWEYS directly competes with DNA for antibody binding and in the other DWEYS binds high-mobility group box-1 and blocks its interaction with RAGE. Our data highlight distinct pathways involved in nucleic acid enters monocytes in SLE, and identify a potential therapeutic to prevent nucleic acid internalization in SLE.

## Introduction

Innate immunity, an evolutionarily conserved branch of the human immune system, employs pattern recognition receptors (PRRs) to recognize pathogen associated molecular patterns (PAMPs), molecules prevalent among bacteria and other microbes, and danger associated molecular patterns (DAMPs), self-molecules that are present at high concentration during tissue injury. PAMPs and DAMPs induce production of immune mediators such as proinflammatory cytokines to promote an inflammatory response. Because PRRs can be activated by host molecules, there are mechanisms to limit access of DAMPs to PRRs. For example, because the DNA sensing Toll-like receptor 9 (TLR9) has an endosomal location, circulating nucleic acid must be internalized to gain access to the endosome to mediate an inflammatory response. The mechanisms that allow such internalization to occur have been described. High-mobility group box-1 (HMGB1)-DNA complexes are internalized following interaction with the RAGE (1, 2) and IgG-DNA immune complexes (ICs) are internalized by Fc receptor engagement (3). Normally, serum levels of HMGB1 are low and IgG anti-DNA antibodies are not present, precluding the entry of extracellular DNA into cells and its transport to the endosomal compartment. While other mechanisms for cellular delivery of nucleic acid have been proposed (4, 5), their significance within the context of autoimmune disease is still not clear.

A member of the Ig superfamily family, RAGE plays a significant role in innate immune signaling (6, 7) and has been shown to recognize several proinflammatory ligands, such as S100 and HMGB1 (8–10). The interaction of HMGB1 with RAGE is of particular interest since it has been shown to be essential for TLR9 activation by nucleic acids (1). RAGE has been implicated in numerous diseases including diabetes, vascular and myocardial disease, Alzheimer's disease, as well as autoimmune diseases such as SLE (7, 9, 11). Since serum levels of DNase I are decreased (12–14) and HMGB1 are increased in SLE (15, 16), the interaction between DNA, HMGB1 and RAGE may have important implications in the pathophysiology of the disease (17).

Existing data also suggest that the Fc receptor II a (FcRIIa) is important for the interferon response observed in SLE, as it functions to internalize ICs composed of DNA-IgG (3). In humans, the FcRII sub-family consists of the pro-inflammatory FcRIIa and the inhibitory FcRIIb, both composed of a single transmembrane chain containing an immunoreceptor tyrosine-based activation motif (ITAM) or immunoreceptor tyrosine-based inhibition motif (ITIM), respectively (18). When FcRIIa on monocytes and monocyte-derived cells binds IgG ICs, the interaction results in downstream inflammatory signaling through activation of the ITAM motif and transport of IgG bound antigen, such as DNA or RNA, to endosomal TLRs. Suppressing FcRIIa expression or blocking its binding to IgG IC results in significant inhibition of type 1 interferon production by healthy monocytes exposed to serum from SLE patients (3). Furthermore, the secretion of type 1 interferon and the subsequent interferon signature is dependent on the nucleic acid component of these ICs interacting with endosomal TLRs (3).

Chronically elevated type 1 interferon and type 1 interferon-induced gene transcripts have been associated with SLE disease activity [see reviews (19, 20)]. Moreover, the induction of type 1 interferon by nucleic acid containing ICs in SLE serum engaging endosomal TLR7 and 9, has been well-documented (20).

While plasmacytoid dendritic cells (pDCs) are established as a source of high secretion of type I interferon both in inflammation and in infection for stimuli (including DNA and RNA viruses as well as synthetic agonists of TLR7 and TLR9) (21), they become refractory to further stimulation and mature to an antigen presenting phenotype. They are also massively outnumbered by the monocytes in blood, especially in active lupus (22, 23). In addition, for some types of stimulation, such as Sendai virus, the monocytes are the primary source of interferon alpha (24). Moreover, for the pristane-induced model of SLE monocytes are the key producers of type I interferon (25). For these reasons we choose to study pathways of type I interferon production in human monocytes. In the current study, pDCs were not tested.

While screening for antigens bound by anti-DNA antibodies, our laboratory identified the DWEYS peptide as a binding target of R4A, a pathogenic mouse monoclonal anti-DNA antibody (26). Subsequently, it was found that antibodies with this cross-reactivity (DNA and DWEYS) are present in approximately 30% of SLE patients (27–31). *In vitro* experiments demonstrate that DWEYS inhibits binding of anti-DNA antibodies to DNA (26, 32, 33) and *in vivo* administration of DWEYS has been shown to protect serologically positive mice from renal deposition of antibody (26, 32). Since the suggested mechanism of action for DWEYS is competitive binding to the antigen binding site of anti-DNA antibodies, we hypothesized that DWEYS might be able to inhibit the proinflammatory effects of DNA-containing ICs.

## Materials and Methods

### Reagents

Sera from active SLE patients or healthy controls were obtained with approval of the Institutional Review Board of the Feinstein Institute for Medical Research. All patients provided written consent for participation in this study. Sera were tested for anti-DNA reactivity and then heat inactivated at 56°C for 30 min and aliquoted for future use. Recombinant HMGB1 (Calmodulin Binding Protein Epitope, Cbp tagged) and reduced HMGB1 was generated as previously described (34, 35). Anti-CD32 monoclonal antibody (clone IV.3) was purchased from STEMCELL Technologies (Cambridge, MA, USA). RAGE and CD32 (FcRIIa) siRNA were purchased from Thermo Fisher Scientific (Waltham, MA, USA). Human recombinant sRAGE was purchased from ProSpec (East Brunswick NJ, USA). DWEYS, biotinylated DWEYS and control peptide (sequence: TATAT) were synthesized by GenScript (Piscataway Township, NJ, USA). FITC labeled and unlabeled CpG oligonucleotides and gardiquimod were purchased from Invivogen (San Diego, CA, USA). AlexaFluor labeled human IgG was purchased from Invitrogen (Carlsbad, CA, USA). AlexaFluor 594 labeled HMGB1 was prepared using Invitrogen reagents according to manufacturer's procedure. Syto 60 DNA reactive dye was purchased from Thermo Fisher Scientific. Calf thymus DNA was purchased from Sigma (St. Louis, MO, USA). DNA was sonicated to produce fragments of approximately 2 kb. Human IgG was heat aggregated as previously described (36).

Purified proteins and culture reagents were endotoxin tested (<0.1 EU/ml) using a Limulus Amebocyte Lysate (LAL) assay kit following the manufacturer's instructions (Lonza. Morristown, NJ, USA).

### Mice

RAGE deficient mice were a courteous gift of Dr. Kevin Tracey. All animal procedures were approved by the Feinstein Institutional Animal Care and Use Committee. Mice were housed in the animal facility of the Feinstein Institute for Medical Research under standard temperature and light and dark cycles.

### Monocyte Isolation and Stimulation

Human cells were obtained with approval of the Institutional Review Board of the Feinstein Institute for Medical Research. PBMCs were isolated from blood of healthy donors (New York Blood Center) by density centrifugation. Monocytes were enriched by negative selection using a human monocyte enrichment kit (STEMCELL Technologies). Purity of monocytes (CD11b^+^, CD14^+^, LAIR-1^+^) was determined to be >90% by flow cytometry (FACS Calibur, BD Biosciences, San Jose, CA, USA). Purified monocytes (2 × 10^6^ cells/ml) were cultured in U-bottom 96-well plates in X-VIVO 15 serum free medium (Lonza) with the indicated reagents and harvested at 4–6 h later, or at the indicated times. Splenic murine monocytes from wild type mice or RAGE deficient mice were isolated using EasySep mouse monocyte enrichment kit (STEMCELL Technologies). Murine monocyte purity was determined to be >90%.

### Quantitative Real Time PCR (qRT-PCR) Analysis

Total RNA was extracted from cells (1–2 × 10^6^ cells per sample) with an RNeasy kit (Qiagen, Germantown, MD, USA) and cDNA was generated using an iScript cDNA synthesis kit (Bio-Rad, Hercules, CA, USA). qRT-PCR was performed on a Light Cycler 480 II (Roche, Indianapolis, IN, USA). Primers for IFIT1 (Hs03027069), IFI44 (Hs00197427), RAGE (Hs00542584), FcRIIa (Hs01013401), MX1 (Hs00182073,), HPRT1 (Hs99999909), and Polr2a (Hs00172187) were purchased from Thermo Scientific. The genes of interest were normalized to the expression of the house keeping gene (Polr2a) and were compared to a control condition with no treatment. The relative induction was calculated by 2^−ΔΔ*Ct*^.

### Transfection

For RNA interference assays, human monocytes (5 × 10^6^ cells) were transfected with RAGE (Thermo Fischer Catalog # AM16708 ID: 110857, 200 nM) or CD32 siRNA (Thermo Fischer Catalog #4392420 ID: s194407, 200 nM) using an Amaxa Nucleofector I Device (Lonza). Transfection efficiency was >40%. The efficiency of the knockdown was determined by immunofluoresence microscopy and q-PCR. Following transfection, cells were cultured for 48 h in 24 wells tissue culture plates in X-VIVO 15 culture medium, and then washed and incubated with indicated under indicated conditions.

### Immunofluorescence Microscopy

For HMGB1 or CpG internalization assays, isolated human monocytes were cultured in 8 well-chamber-slides (Nunc, LabTek, Thermo-Fisher) in X-VIVO 15 culture medium. Cells were allowed to adhere for 2 h and then washed with PBS and incubated with AlexaFluor-594 HMGB1 or FITC-CpG with or without DWEYS for 15 min. Nuclei were counterstained using DAPI. Cells were washed three times with cold PBS and then fixed with 4% PFA and mounted using Dako mounting solution (Agilnet Technologies, Santa Clara, CA, USA).

### DWEYS Internalization Experiments

Primary human monocytes were incubated for 20 min with biotin-tagged DWEYS peptide alone or with serum from SLE patients or healthy controls. Following incubation, cells were thoroughly washed and stained with anti-CD14 antibody (R&D Technologies, North Kingston, RI, USA) for 40 min at 4°C. Cells were then washed, fixed using 4% PFA and permeabilized using 1% Triton-X100, followed by incubation with labeled streptavidin for 40 min. Cell nuclei were counterstained with DAPI and cells were imaged using an ImageStream device (Amnis, Burlington, MA, USA).

### Binding Studies

Binding of TLR9 to biotinylated CpG or the DWEYS peptide was assayed in a buffer consisting of 50 mM Tris-HCl pH 7.4, 100 mM NaCl, 1% ultrapure BSA, 0.01% Tween 20 using an amplified luminescent proximity assay with streptavidin-conjugated donor beads and nickel chelate acceptor beads (AlphaScreen; Perkin Elmer, Boston, MA, USA), as described before (1).

### Surface Plasmon Resonance (SPR) Analysis

A Biacore T200 apparatus (GE Healthcare Bio-Sciences, Pittsburgh, PA, USA) was used for real-time binding interaction studies. For HMGB1 and DWEYS peptide binding analyses, HMGB1 protein was immobilized onto a CM5 series chip (GE Healthcare). The HMGB1 protein was diluted to a concentration of 20 μg/mL in 10 mM sodium acetate buffer (pH = 4.5). A 1:1 mixture of N-hydroxysuccinimide and N-ethyl-N-(dimethyaminopropyl) carbodiimide was used to activate two flow-cells of the CM5 chip. One flow-cell was used as a reference and thus immediately blocked upon activation by 1 M ethanolamine (pH = 8.5). The sample flow-cell was injected with the diluted HMGB1 at a flow rate of 10 μL/min. The HMGB1 injection was stopped when the surface plasmon resonance reached ~600 RU. For HMGB1 and DWEYS peptide kinetics assay, DWEYS peptide were sequentially injected at a flow rate of 20 μL/min for 120 s at 25°C, the dissociation time was set for 2 min. The concentrations were 16, 31.25, 62.5, 125, 250 μM. The equilibrium dissociation constant (KD) was obtained to evaluate the binding affinity by using the BIAEvaluation 2.0 software (GE Healthcare) assuming a 1:1 binding ratio. At least 3 independent experiments were performed.

For sRAGE/HMGB1 inhibition assay, human recombinant sRAGE was immobilized onto a CM5 series chip (GE Healthcare). The sRAGE protein was diluted to a concentration of 20 μg/mL in 10 mM sodium acetate buffer (pH = 4.5). A 1:1 mixture of N-hyrdoxysuccinimide and N-ethyl-N-(dimethyaminopropyl) carbodiimide was used to activate two flow-cells of the CM5 chip. One flow-cell was used as a reference and thus immediately blocked upon activation by 1 M ethanolamine (pH = 8.5). The sample flow-cell was injected with diluted sRAGE at a flow rate of 10 μL/min. The sRAGE injection was stopped when the surface plasmon resonance reached ~500 RU. The recombinant HMGB1 (0.5 μM) with or without DWEYS peptide used in serial dilutions from 50 to 1.6 μM was injected over sRAGE immobilized chip at a flow rate of 30 μL/min for 60 s at 25°C. The dissociation time was set for 1 min. Binding experiments were conducted in PBS + 0.05 Tween 20 as the running buffer. At least 3 independent experiments were performed.

### Serum DNA Displacement

Calf thymus double stranded (ds)DNA was incubated for 1 h with Syto 60 DNA dye. DNA was then dialyzed overnight to remove free dye. Serum of active SLE patients with anti-DNA reactivity was incubated for 1 h with Syto 60 labeled DNA, and then for 1 h with DWEYS or control peptide, followed by IgG isolation using protein G beads (Thermo Fisher) as per manufacturer's instructions. Presence of Syto 60 labeling (652/678 absorption, emission maxima) was evaluated using 700 nm channel of Odyssey scanner (LI-COR Biosciences, Lincoln, NE, USA).

### Statistical Analysis

Numerical values represent the mean of at least three independent experiments in which each condition was tested in triplicate. Variance of mean values between two groups was analyzed by the student's *t*-test for unpaired observations. Group differences were tested with one-way ANOVA followed by Bonferroni's correction for multiple comparisons. ^*^P < 0.05, ^**^P < 0.01, ^***^P < 0.001. Statistical analyses were performed using Prism 6 (Graphpad Software, La Jolla, CA, USA).

## Results

### The Interferon Signature Is Mediated Through Both IgG:FcR and HMGB1:RAGE Interactions

**Figure 1:**
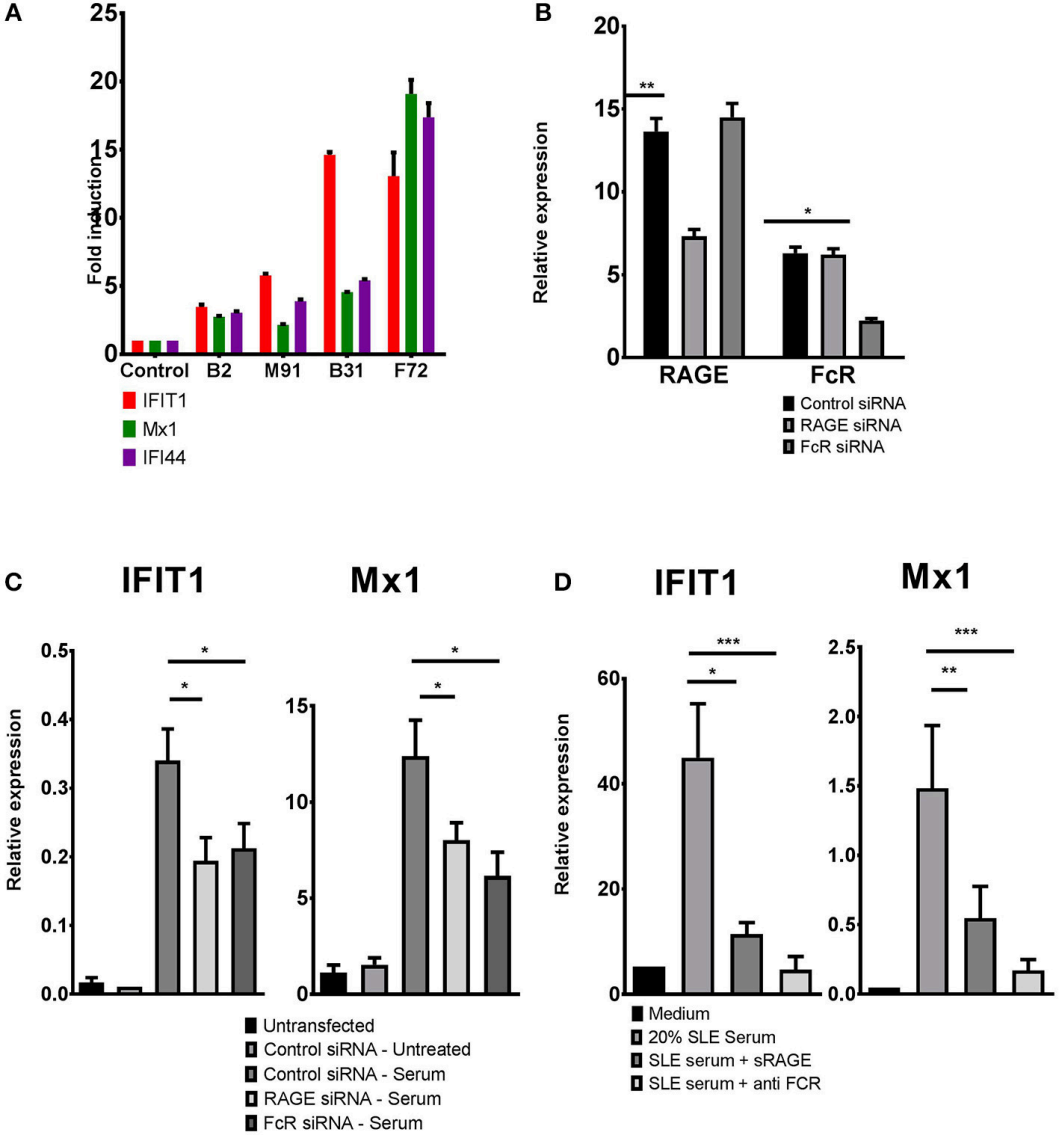
ISG induction by SLE serum requires either RAGE or FcRlla. **(A)** Primary human monocytes were incubated with serum from different SLE patients, B2, M91, B31, F72, or control sera, for 4 h. ISG expression was evaluated using qPCR. Data plotted represents fold induction for each gene relative to the sample incubated with control serum and is representative of 30 samples tested. **(B)** RAGE and FcRlla levels in monocytes treated with RAGE or FcRIIa siRNA, assessed by qPCR. **(C)** Monocytes were transfected with RAGE, FcRlla, or control siRNA, prior to incubation with SLE serum for 4 h. For each sample (control, RAGE, or FcRlla siRNA transfected cells) fold induction of ISGs was calculated as the ratio of gene expression between treated and untreated cells. **(D)** Primary human monocytes were treated with SLE serum, with or without sRAGE or monoclonal anti-FcRlla antibody for 4 h. ISG expression was analyzed by qPCR. Results in each case **(B–D)** indicate mean ± SD of three independent experiments. ^*^p < 0.05; ^**^p < 0.01; ^***^p < 0.001.

Total blood cells or PBMCs of active SLE patients often show increased levels of interferon stimulated genes (ISGs), known as the “interferon signature” (37). Due to the technical challenges in measuring type 1 interferon levels, ISG expression often is used an as indicator of interferon activity. It has been shown that the interferon signature is transferable to healthy PBMCs by incubation with SLE serum and is dependent on nucleic acid containing ICs (3, 38). We incubated blood monocytes from healthy donors with control serum or serum from SLE patients, and confirmed that healthy monocytes showed ISG upregulation when incubated with SLE serum consistent with increased TLR activation, whereas incubation of healthy monocytes with serum from control, healthy donors, did not exhibit ISG upregulation (Figure 1A). Figure 1A shows representative data of the induction of ISG in normal blood monocytes with healthy control serum and with four lupus sera: B2, M91, B31, and F72. The degree of induction of ISGs was variable, and about 6 of 30 SLE serum samples tested failed to induce ISG upregulation, consistent with data showing that 60–80%, but not all, SLE patients, exhibit an increased activation of the type 1 interferon pathway (20). We selected only positive sera for further studies. Of the 24 positive sera tested, 22 showed a 25% or more inhibition with DWEYS peptide.

A mechanism of induction of the interferon signature in SLE has been shown to be TLR activation by ICs containing nucleic acid. Since it has been demonstrated, however, that DNA can access TLR9 through a FCRIIa mediated mechanism or through an HMGB1-RAGE dependent mechanism, we sought to determine which of these was responsible for the induction of an interferon signature by SLE serum.

We addressed this by using siRNA to knock down RAGE or FcRIIa in primary human monocytes. Following transfection with siRNA, expression of either RAGE or FcRIIa was decreased by about 50% (Figure 1B). Compared to cells treated with control siRNA, cells treated with either RAGE or FcRIIa siRNA showed a significantly reduced induction of ISGs (IFIT1 and Mx1) when incubated with SLE serum (Figure 1C and Supplementary Figure 1). These results were confirmed using a soluble form of RAGE (sRAGE) acting as a decoy receptor and an antibody to FcRIIa, to block RAGE or FcRIIa mediated activation. Again, cells treated with either sRAGE or anti-FcRIIa antibody showed a significantly reduced induction of either IFIT1 or Mx1 following incubation with SLE sera, confirming that activation of either RAGE or FcRlla by SLE serum can contribute to the induction of ISGs in healthy monocytes (Figure 1D).

### DWEYS, a Peptide Mimetope of DNA, Inhibits Induction of ISGs

We have previously shown that a subset of anti-dsDNA antibodies cross-reacts with the pentapeptide DWEYS (26, 32, 33). Since DWEYS has been shown to inhibit IC-mediated glomerular damage by preventing the binding of antibody to tissue (26, 32), we hypothesized that DWEYS as a DNA mimetope might also be able to block the transfer of the interferon signature, by binding TLR9 and blocking its activation by DNA, thereby functioning as a TLR9 antagonist.

**Figure 2:**
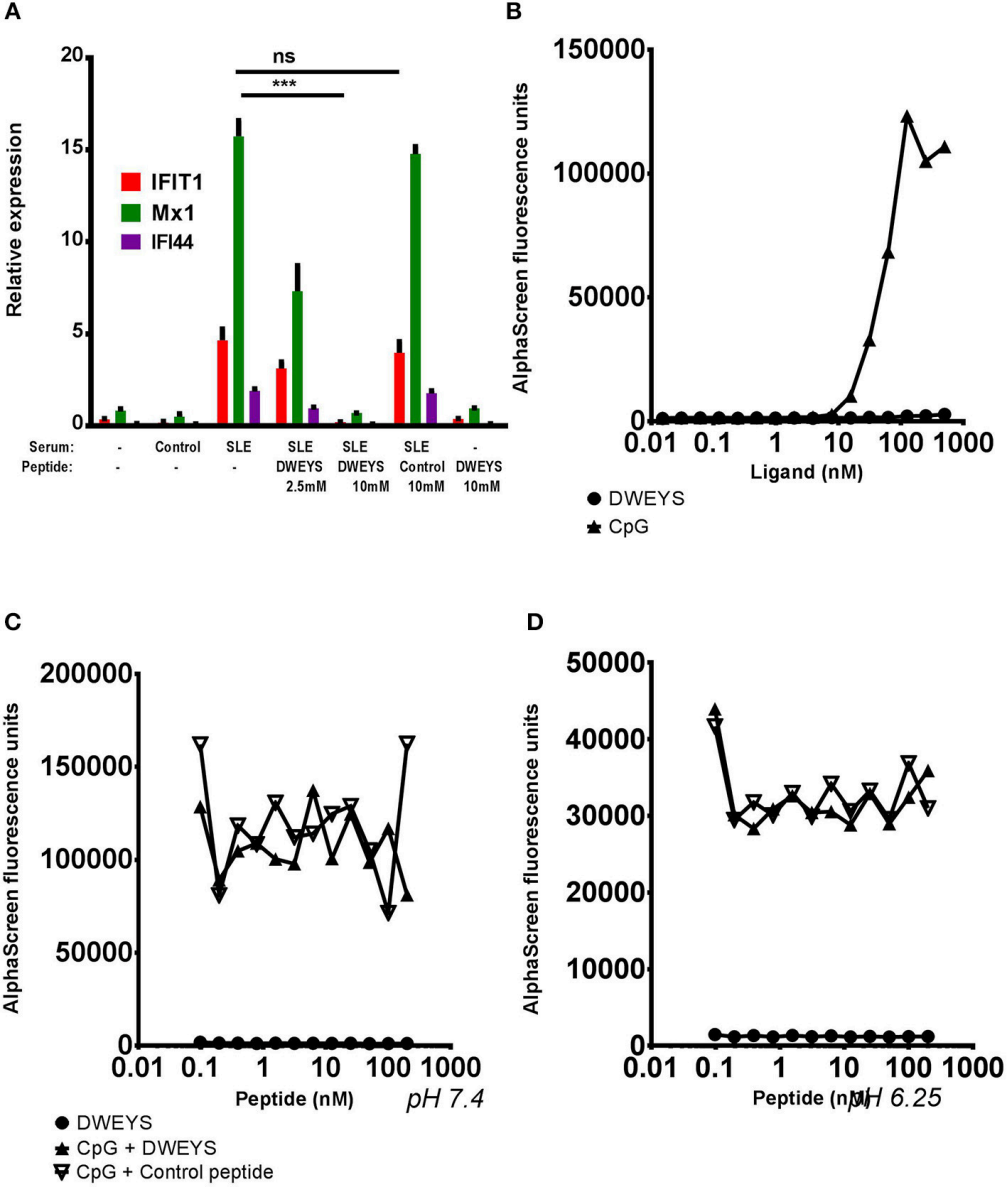
DWEYS inhibits the transfer of the interferon signature by SLE serum but is not a TLR9 antagonist. **(A)** Primary human monocytes were incubated with SLE serum with or without DWEYS or with control peptide for 4 h. lSG expression was evaluated by qPCR. Results indicate mean ± SD of three independent experiments. **(B)** Assessment of DWEYS and CpG oligonucleotides binding to TLR9. Labeled TLR9 was incubated with varying concentrations of CpG or DWEYS for 30 min in room temperature. Binding was assessed using an AlphaScreen assay as described in the methods section. **(C,D)** Inhibition assay. Labeled TLR9 and constant, binding concentration of CpG were incubated with different concentrations of DWEYS or a control peptide at neutral (pH 7.4, **C**) or acidic (pH 6.25, **D**) condition. Binding was assessed using an AlphaScreen assay as noted in the methods section. Results are representative of three independent experiments. ^***^p < 0.001, ns, not significant.

To test this hypothesis we incubated monocytes from healthy donors with SLE or control serum together with two different concentrations of DWEYS or with the higher dose of an unrelated control peptide (Figure 2A). While monocytes incubated with SLE serum, with or without control peptide, showed an induction of all three ISGs studied, DWEYS peptide significantly inhibited this activation (Figure 2A). Notably, even though DWEYS is a DNA mimetope it failed to produce an interferon response on its own even at the highest range of concentrations tested (see Figure 2C).

Since DWEYS blocked induction of an interferon signature, we also asked whether it could potentially function as a TLR9 antagonist. To address this possibility we first asked whether DWEYS could directly bind to TLR9. The DWEYS peptide showed negligible binding to immobilized TLR9, at all concentrations tested, compared to CpG oligonucleotide (CpG) that bound TLR9 in a dose-dependent fashion in an AlphaScreen assay (Figure 2B). Furthermore, neither DWEYS, nor a control peptide, used at various concentrations, inhibited the binding of a constant concentration of CpG to TLR9 (Figures 2C,D) in both at neutral, or acidic pH (simulating activated endosome). Thus, we concluded that DWEYS was functioning upstream of TLR9.

### DWEYS Does Not Prevent Internalization of Aggregated IgG

**Figure 3:**
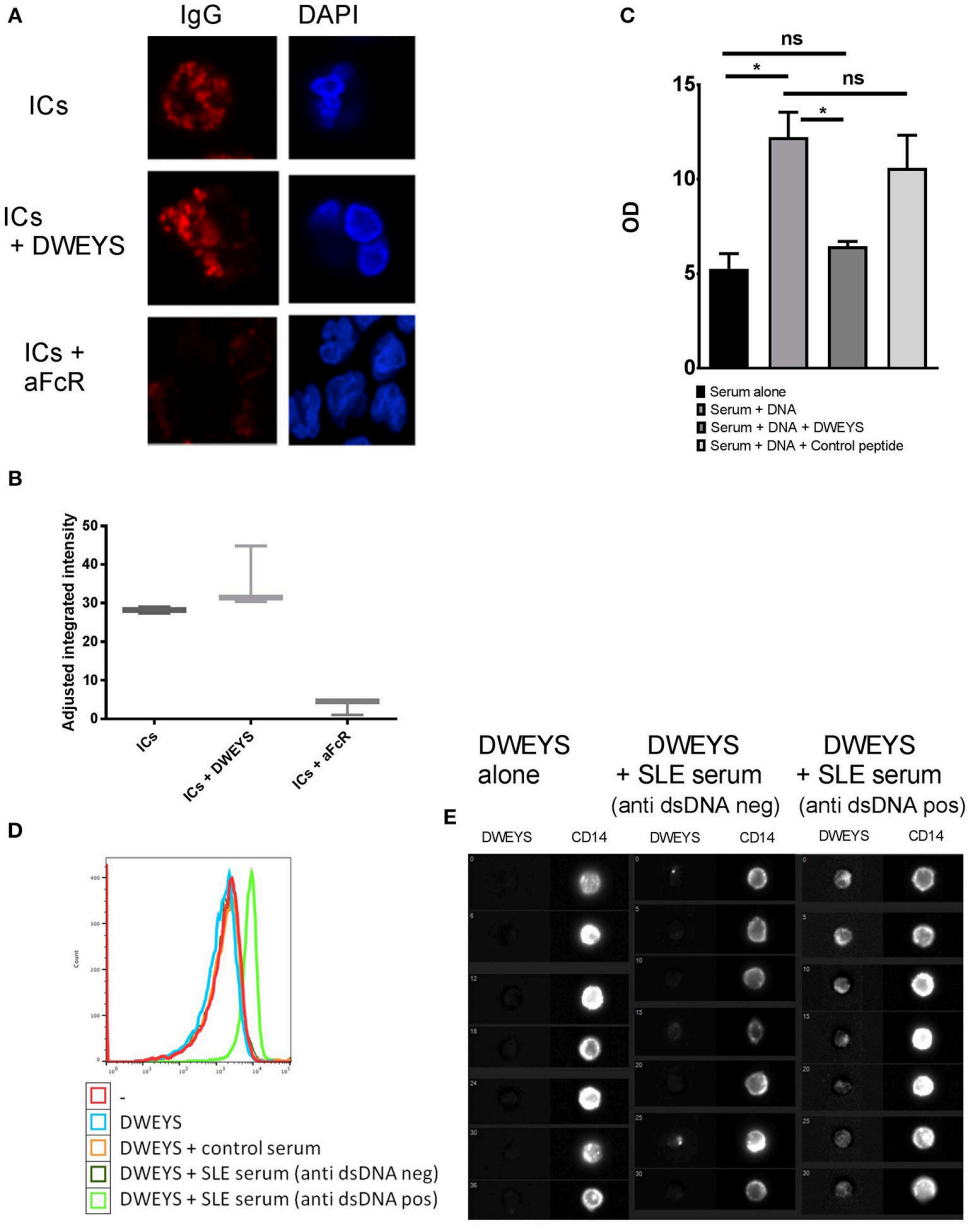
DWEYS displaces DNA from immune complexes and inhibits DNA internalization into monocytes. **(A)** Primary human monocytes were incubated with labeled heat aggregated human lgG (hlgG, red) and either DWEYS or a blocking anti-FcRIIa antibody for 20 min. Cells were counterstained with DAPI and imaged using a laser scanning confocal microscope. **(B)** The graphs indicates adjusted fluorescence intensity for three experiments. **(C)** SLE serum was incubated with Syto 60 labeled dsDNA (5 μM), followed by DWEYS or control peptide (100 μM) for 30 min each. lgG was then isolated on protein G beads and Syto60 levels from protein G beads were quantified using LICOR scanner. OD was normalized to a standard labeled and filtered DNA. Results indicate mean ± SD of three independent experiments. **(D,E)** Primary human monocytes were incubated with biotinylated DWEYS alone or biotinylated DWEYS and SLE serum from patients with or without anti ds DNA antibodies for 20 min. Cells were then stained for CD 14 as a membrane marker and intracellular DWEYS was tagged using fluorochrome labeled streptavidin. Intracellular DWEYS was quantified using lmageStream instrument: DWEYS fluorescence levels by flow cytometry **(D)** and representative ImageStream cell images **(E)**. One representative experiment of three with similar results is shown. ^*^p < 0.05; ns, not significant.

Because DWEYS was not an antagonist of TLR9, we asked whether DWEYS could inhibit FcRlla-mediated uptake of ICs. FITC labeled human IgG antibodies were heat aggregated to simulate ICs with respect to FcR binding and then incubated with monocytes from healthy donors. After 30 min of incubation, cytoplasmic IgG could be clearly identified, showing an endosomal staining pattern (Figure 3A, top panel). Internalization was greatly diminished following the addition of a blocking anti-FcRIIa antibody (Figure 3A, bottom panel), supporting previously published data showing that internalization of IgG ICs is dependent upon FcRIIa (3). In contrast to these data, addition of DWEYS resulted in no inhibition of antibody internalization, demonstrating that DWEYS does not inhibit internalization of IgG aggregates into monocytes (Figure 3A, middle panel). Figure 3B depicts quantification of the data.

Since DWEYS did not inhibit the internalization of IgGs, we tested whether it could displace DNA from ICs in SLE serum. Serum from SLE patients with active disease and a high titer of anti-DNA antibodies was incubated with Syto 60 labeled dsDNA, and then with either DWEYS or control peptide. Following incubation, serum IgG was isolated using protein-G beads, and Syto 60 signal of the IgG-bound beads was quantified using a LI-COR infra-red scanner. dsDNA was readily detected in isolated IgG, and the addition of DWEYS significantly decreased the amount of DNA detected, Figure 3C. We, therefore, concluded that DWEYS was able to competitively inhibit the incorporation of DNA into ICs. To further investigate whether DWEYS was able to incorporate into ICs and displace dsDNA, we incubated monocytes with labeled DWEYS, with or without serum from SLE patients. We used serum with or without elevated titers of anti-DNA antibody. Monocytes were also incubated with serum from healthy controls. Monocytes were then assessed for internalization of DWEYS using an ImageStream instrument (Figures 3D,E). ImageStream technology allows for incorporation of microscopy into conventional flow cytometry. Thus, cellular localization of the labeled DWEYS could be evaluated. Intracellular DWEYS was determined by defining a membrane mask with CD14 labeling. DWEYS was considered to be intracellular in regions interior to the membrane mask. Free DWEYS, as well DWEYS in the presence of serum from healthy donors, showed only minimal internalization (Figure 3D). In contrast, in the presence of SLE serum from patients with high anti-DNA antibody titer, significant intracellular internalization of DWEYS was seen (Figure 3D). To affirm the hypothesis that DWEYS internalization by monocytes was dependent upon the presence of anti-DNA antibodies in the serum, we repeated the experiment with serum from active SLE patient with low titers of anti-DNA antibodies. As expected, serum lacking anti-DNA antibodies did not mediate DWEYS internalization (Figure 3D). Figure 3E depicts these data assessed by flow cytometry.

The induction of ISGs by the TLR7 agonist, gardiquimod, is also DWEYS inhibitable (Supplementary Figure 2). Thus, it seems that DWEYS peptide has the capacity to block the stimulation of both, TLR9 and TLR7.

### DWEYS Blocks the HMGB1:RAGE Pathway

**Figure 4:**
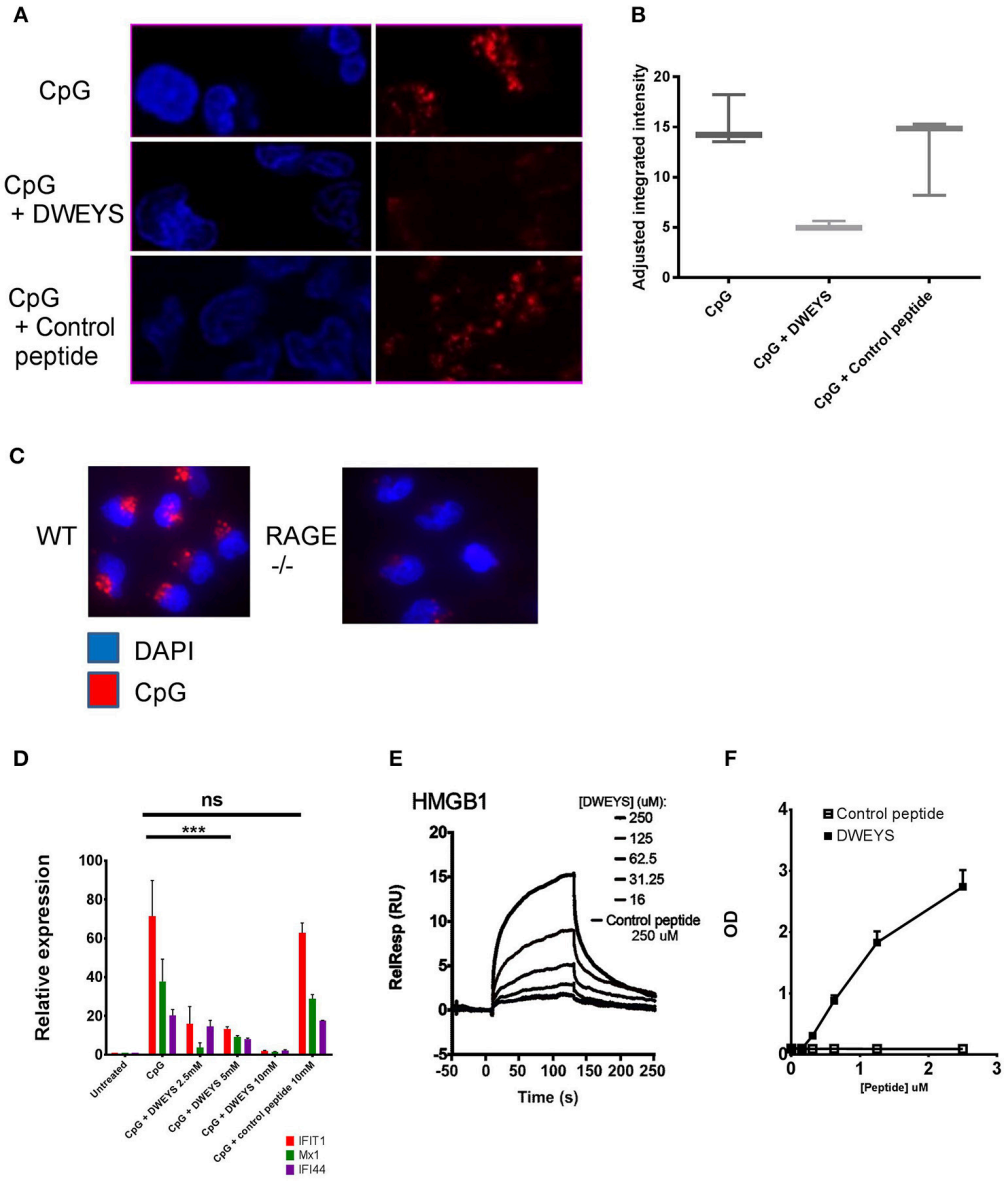
DWEYS binds HMGB1 and inhibits its binding to RAGE. **(A)** Monocytes were incubated with labeled CpG (red) and DWEYS or control peptide for 20 min. Cells were counterstained with DAPI prior to imaging. **(B)** Depicts adjusted fluorescence intensity for three experiments run. **(C)** Monocytes from C57BL6 wild type (WT) or RAGE^−/−^ mice were incubated with labeled CpG for 20 min and counterstained with DAPI prior to imaging. **(D)** Primary human monocytes were incubated with CpG and with or without DWEYS or a control peptide for 20 min. ISG expression was analyzed by qPCR. Data were plotted relative to untreated cells, and represents three independent experiments. **(E,F)** HMGB1-DWEYS binding analysis by biacore T200 **(E)** and ELISA **(F)**. HMGB1 was immobilized on a CM5 sensor chip and varying peptide concentrations were injected as noted in the methods section. DWEYS binds to HMGB1 with an apparent KD of 185 μM. ^***^p < 0.001; ns, not significant.

Non IC-bound DNA can also be internalized by binding to HMGB1, with the HMGB1-DNA complex binding to RAGE (1). Indeed, HMGB1 bound to circulating nucleosomes, has been shown to induce an inflammatory response in SLE (39) as well as HMGB1 ICs (40). We, therefore, asked whether DWEYS could also inhibit the intracellular delivery of HMGB1-bound nucleic acid. We incubated monocytes from healthy donors with FITC labeled CpG. At 37°C, cellular internalization of FITC-CpG was evident after 15 min of incubation (Figure 4A, top panel). Addition of DWEYS led to a significant decrease in CpG internalization (Figure 4A, middle panel) while a control peptide had no effect (Figure 4A, bottom panel). Figure 4B shows quantification of the data. Consistent with previous reports (1), CpG failed to be internalized in monocytes derived from spleens of RAGE^−/−^ mice, confirming that CpG is internalized in a RAGE dependent pathway (Figure 4C). To further demonstrate that DWEYS was able to inhibit the internalization of DNA by monocytes, we evaluated ISG induction in CpG exposed monocytes with or without DWEYS, and showed that DWEYS inhibited CpG induction of ISGs in a dose-dependent manner, while the highest concentration of a control peptide failed to inhibit ISG induction (Figure 4D). CpG is internalized chaperoned by HMGB1 binding through RAGE. Monocyte activation by CpG could be suppressed by soluble RAGE and not by anti-FcRlla antibody (Supplementary Figure 1A). Treatment of monocytes with RAGE siRNA but not control siRNA also diminished ISG induction (Supplementary Figure 1B), confirming that CpG activates monocytes through a RAGE-dependent pathway.

Because it is known that nucleic acid is transported into cells by HMGB1 in a RAGE dependent fashion, we asked whether DWEYS could bind HMGB1. In a surface plasmon resonance assay, recombinant HMGB1 was immobilized on an assay chip and different concentrations of DWEYS or control peptide were assessed for binding to immobilized HMGB1. DWEYS exhibited dose related binding to HMGB1, while control peptide did not bind (Figure 4E). The equilibrium constant, KD, of HMGB1 and DWEYS was calculated to be 185 μM. Similar results were obtained by ELISA with HMGB1-coated plates and increasing concentrations of DWEYS (Figure 4F).

**Figure 5:**
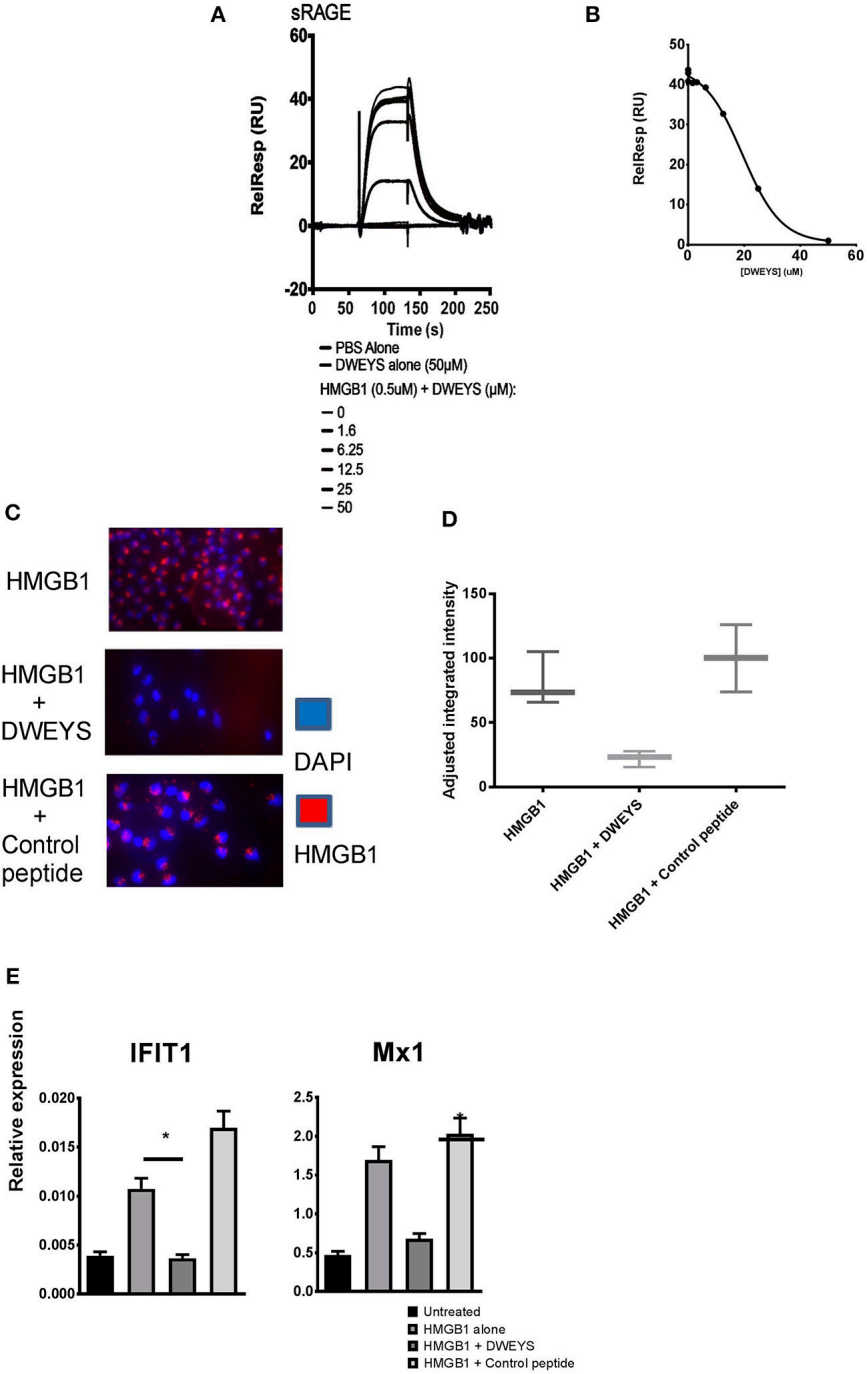
**(A)** Biacore T200 analysis of DWEYS inhibition of HMGB1-RAGE binding. sRAGE was immobilized on a CM5 sensor chip, HMGB1 with or without varying concentrations of DWEYS were added as analytes. IC50 was 34.7 μM. **(B)** Inhibition curve used for IC50 evaluation as noted in the methods section. **(C,D)** Human monocytes were incubated with labeled HMGB1 (red) alone (**C**, top panel) or HMGB1 and DWEYS (**C**, middle panel) or HMGB1 and control peptide (**C**, bottom panel) for 20 min. Cells were counterstained with DAPI and imaged using a confocal laser scanning microscope. **(D)** Indicates adjusted fluorescence intensity for three experiments. **(E)** Primary human monocytes were incubated with HMGB1 and with or without DWEYS or control peptide for 4 h. lSG expression was evaluated using qPCR. Results indicate mean ± SD of three independent experiments. ^*^p < 0.05.

As previously reported, HMGB1 chaperones nucleic acids to toll like receptors in a RAGE dependent pathway (2). We, therefore, asked whether binding of HMGB1 to DWEYS would affect its ability to bind RAGE. Again, using surface plasmon resonance assay, DWEYS significantly inhibited the binding of HMGB1 to RAGE in a dose-dependent manner (Figure 5A). Figure 5B depicts the inhibition curve, with half maximal concentration (IC_50_) of 34.7 μM. Because HMGB1 is internalized into monocytes through RAGE, we asked whether HMGB1 would penetrate monocytes in the presence of DWEYS. AF-594-labeled HMGB1 was detected in monocytes after 15–30 min in incubation (Figure 5C, top panel), but failed to be internalized when DWEYS was present (Figure 5C, middle panel). A control peptide displayed no inhibition of HMGB1 internalization (Figure 5C, bottom panel). Figure 5D shows quantification of the data. Consistent with these observations, monocytes incubated with HMGB1 showed an increased level of ISGs, in the presence of control peptide but failed to do so in the presence of DWEYS (Figure 5E).

## Discussion

The interferon pathway is an established contributor to disease in some SLE models (41). This observation, together with association of an interferon signature with active disease SLE in patients (20), has led investigators to query the contribution of interferon to SLE pathogenesis by making it a target for disease modifying drugs (42). Despite the compelling rationale for inhibiting interferon, trials using monoclonal anti-interferon antibodies have not shown therapeutic benefit in SLE patients, despite inhibiting the interferon signature. Trials of antibodies directed against the type-1 interferon receptor (IFNaR) appear more promising; however, their efficacy is not yet fully determined (42). It is clear, however, that an increased risk of viral infections occurs with interferon targeted treatments, and that interferon-based therapies are not without risk.

The interferon signature is induced in SLE by a number of mechanisms, by interferon itself, and by RNA or DNA-containing immune complexes with subsequent activation of TLR7 and 9. While most effort has been concentrated on assessing interferon or interferon receptor blockade, there are also TLR antagonists under development (43). In some mouse models of SLE, TLR inhibition has resulted in a steroid-sparing effect and a favorable effect on disease progression (44). However, while TLR7 and 9 activation induces interferon, it also induces NF_K_B-dependent cytokines. Therefore, blocking the TLR pathway upstream of interferon would have a broader anti-inflammatory effect than blocking interferon alone. Moreover, while associated with many proinflammatory processes, such as B cell activation (45) and differentiation of pro-inflammatory CD4^+^ T cells and dendritic cells (46), some data suggest that type 1 interferon is protective in some patients and mouse strains (47–51). Thus, a therapy focused on a broader spectrum of inflammatory mediators may exhibit enhanced efficacy and be beneficial even in individuals in whom high interferon levels may represent a compensatory response to inflammation.

In this study, we demonstrate a fourth mechanism for the induction of an interferon signature; HMGB1 binds RAGE in SLE serum and chaperones DNA and RNA to endosomal TLRs. While a previous study showed that soluble RAGE or anti-HMGB1 antibody can inhibit the induction of an interferon signature by SLE serum, the authors assumed that HMGB1 is present in immune complexes (1). Here we show that HMGB1 in SLE serum can directly bind to RAGE facilitating an interferon signature.

We also demonstrate effective targeting of the interferon response mediated by SLE serum using DWEYS peptide. As noted earlier, previously published data have demonstrated that the type 1 interferon response seen in healthy PBMCs by exposure to SLE serum can be driven by DNA containing ICs which are internalized through FcRIIa, and then signal through TLR9 (3). Data from other studies have shown that nucleic acids can be chaperoned to endosomal TLRs by HMGB1 and RAGE and that this may occur in SLE (2). The centrality of RAGE in the transport of nucleic acid into cells was shown by the observation in several studies that neither nucleic acid, nor the DNA binding protein HMGB1, can be internalized in RAGE deficient cells (52–54). There is only one contradictory report demonstrating of CpG-HMGB1 internalization in the absence of RAGE (1). As this was study of dendritic cells (DCs) rather than monocytes, the results may reflect cell specific differences. Interestingly, in that study, the CpG that was internalized through a RAGE-independent pathway did not activate TLR9 to the level of RAGE sufficient activation, suggesting that it did not traffic to an endosomal compartment. Consistent with these observations, non-stimulatory oligonucleotides which bind HMGB1 lead to significant inhibition of the interferon signature in CpG treated DCs (55). Delivery of oligonucleotide:HMGB1 complexes to intracellular compartments were not affected in this model. This is in contrast to our model in which DWEYS peptide blocks binding of DNA:HMGB1 complexes to RAGE, thus, preventing activation of endosomal TLR9 by DNA. Moreover, DWEYS peptide blocks the induction of ISGs by the TLR7 agonist, gardiquimod, as it prevents internalization of HMGB1 and its cargo of TLR agonists.

**Figure 6:**
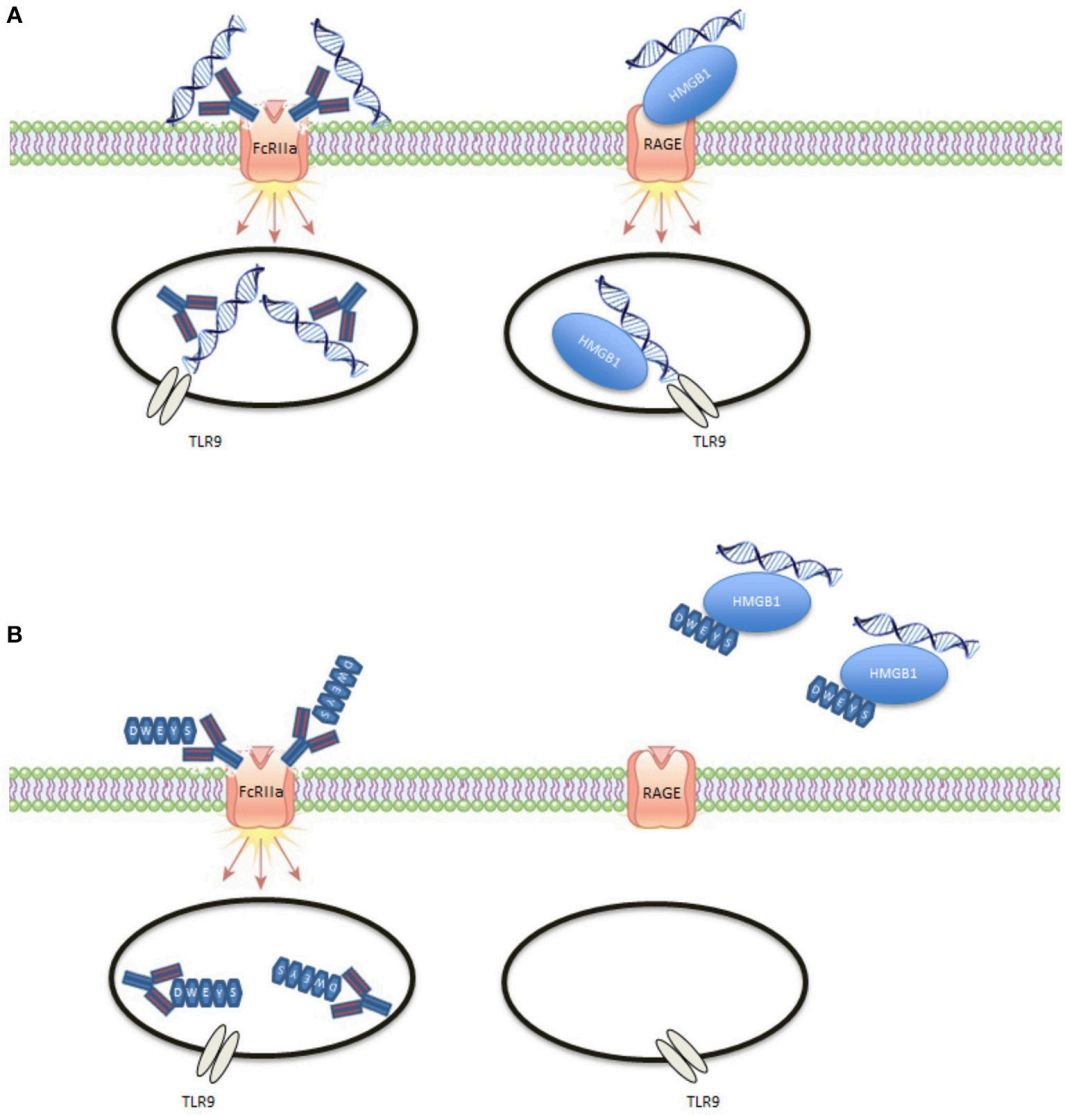
Illustration of DWEYS mediated inhibition. **(A)** DNA can enter endosomes as ICs through FcRlla or complexed to HMGB1 through RAGE. **(B)** DWEYS replaces DNA in DNA containing ICs and blocks the interaction of HMGB1 with RAGE.

Our data build on these studies and confirm two distinct mechanisms by which SLE serum can induce ISGs (Figure 6). In the first pathway, nucleic acid-IgG ICs are internalized into monocytes through FcRIIa. In the second, nucleic acid HMGB1 complexes are internalized through an HMGB1-RAGE interaction. Both pathways signal through TLR9 and result in an interferon response. The second pathway helps explain the basis of an interferon signature in SLE patients with low anti-DNA and anti-RNP antibody titers. It is important to note that for some sera blocking the RAGE pathway seemed to have a less pronounced effect on the interferon signature than blocking the FcRIIa pathway; for other sera, the opposite was true. Sera from patients with high titers of anti-DNA antibodies predominantly triggered the FcRIIa pathway. Blocking both pathways with one blocking agent offers obvious benefits.

Overall, this study demonstrates that activation of endosomal TLRs in SLE, and in particular TLR9, occurs through more than one pathway and involves both IgG bound and HMGB1 bound DNA in SLE. By intercalating into DNA-containing ICs, together with preventing the binding of HMGB1 to RAGE, DWEYS was able to inhibit the internalization of DNA into monocytes by both pathways, thereby preventing downstream activation of TLR9. Since DWEYS was able to block both pathways leading to DNA internalization into monocytes, as well as to block internalization off an RNA homolog, gardiquimod, it can inhibit much of the interferon production triggered by SLE serum. It should however, have no effect on innate immune activation by viral infections in which viral PAMPS are delivered to endosomal compartments by receptor-specific endocytosis and other pathways independent of TLR activation. As a therapeutic, it should not lead to the same increase in viral infections that is seen with anti-interferon therapy. Since HMGB1 itself is implicated in the pathogenesis of SLE, contributing to the generation of anti-DNA antibodies and preventing lymphocyte apoptosis, an intervention that targets the function of HMGB1 might prove to be superior to targeting interferon. Indeed, monoclonal anti-HMGB1 antibodies have been shown to ameliorate polyarthritis and lupus-like disease in mouse models (56, 57). Thus, the DWEYS peptide abrogating TLR9 activation through two pathways may be a candidate therapeutic for SLE.

## Author Contributions

AP, EG, EL, MH, IB-Z, and MS performed the studies. AP, EG, EL, IB-Z, YA-A, and BD designed the studies. AP, EG, CK, YA-A, EL, MS, and BD analyzed data. AP, EG, CK, and BD wrote the manuscript.

### Conflict of Interest Statement

The authors declare that the research was conducted in the absence of any commercial or financial relationships that could be construed as a potential conflict of interest.

## Supplementary Material

Supplementary Figure 1 **(A,B)** ISG induction by CpG is inhibited by blocking RAGE, but not FcRIIa. **(A)** Primary human monocytes were incubated with CpG alone or with soluble RAGE (sRAGE) or with blocking anti-FcRIIa antibody for 4 hours. ISG levels were assessed by qPCR. **(B)** Primary human monocytes were transfected with control or RAGE siRNA as noted in the methods section. Cells were then treated with CpG alone (+) or with DWEYS (+D) for 4 h. IFIT1 levels were assessed by qPCR. Results indicate mean ± SD of three independent experiments. ^*^*P* < 0.05; ^**^*P* < 0.01; ^***^*P* < 0.001.

Supplementary Figure 2 DWEYS inhibited ISG induction by TLR7 agonist, gardiquimod. Primary human monocytes were incubated with gardiquimod alone, or with soluble DWEYS peptide at indicated molar excess for 4 h. ISG levels were assessed by qPCR as in all other experiments.

## Abbreviations

PAMPs: pathogen associated molecular patterns
DAMPs: damage associated molecular patterns
PRRs: pattern recognition receptors
TLRs: toll-like receptors
SLE: systemic lupus erythematosus
FcRIIa, FcRIIb: Fc gamma receptor IIa or IIb
HMGB1: high-mobility group-1
RAGE: advanced glycation end products
qRT-PCR: quantitative real time PCR
ITAM: immunoreceptor tyrosine-based activation motif
ITIM: immunoreceptor tyrosine-based inhibition motif
ISGs: interferon stimulated genes.

## References

[1] Tian J Avalos AM Mao SY Chen B Senthil K Wu H. Toll-like receptor 9-dependent activation by DNA-containing immune complexes is mediated by HMGB1 and RAGE. Nat Immunol. (2007) 8:487–96. 10.1038/ni1457 17417641

[2] Yanai H Ban T Wang Z Choi MK Kawamura T Negishi H. HMGB proteins function as universal sentinels for nucleic-acid-mediated innate immune responses. Nature (2009) 462:99–103. 10.1038/nature08512 19890330

[3] Means TK Latz E Hayashi F Murali MR Golenbock DT Luster AD. Human lupus autoantibody-DNA complexes activate DCs through cooperation of CD32 and TLR9. J Clin Invest. (2005) 115:407–17. 10.1172/JCI23025 15668740 PMC544604

[4] Chamilos G Gregorio J Meller S Lande R Kontoyiannis DP Modlin RL. Cytosolic sensing of extracellular self-DNA transported into monocytes by the antimicrobial peptide LL37. Blood (2012) 120:3699–707. 10.1182/blood-2012-01-401364 22927244 PMC3488884

[5] Lahoud MH Ahmet F Zhang JG Meuter S Policheni AN Kitsoulis S. DEC-205 is a cell surface receptor for CpG oligonucleotides. Proc Natl Acad Sci USA. (2012) 109:16270–5. 10.1073/pnas.1208796109 22988114 PMC3479608

[6] Schmidt AM Yan SD Yan SF Stern DM. The biology of the receptor for advanced glycation end products and its ligands. Biochim Biophys Acta (2000) 1498:99–111. 10.1016/S0167-4889(00)00087-2 11108954

[7] Kierdorf K Fritz G. RAGE regulation and signaling in inflammation and beyond. J Leukoc Biol. (2013) 94:55–68. 10.1189/jlb.1012519 23543766

[8] Leclerc E Fritz G Vetter SW Heizmann CW. Binding of S100 proteins to RAGE: an update. Biochim Biophys Acta (2009) 1793:993–1007. 10.1016/j.bbamcr.2008.11.016 19121341

[9] Sims GP Rowe DC Rietdijk ST Herbst R Coyle AJ. HMGB1 and RAGE in inflammation and cancer. Annu Rev Immunol. (2010) 28:367–88. 10.1146/annurev.immunol.021908.132603 20192808

[10] Hori O Brett J Slattery T Cao R Zhang J Chen JX. The receptor for advanced glycation end products (RAGE) is a cellular binding site for amphoterin. Mediation of neurite outgrowth and co-expression of rage and amphoterin in the developing nervous system. J Biol Chem. (1995) 270:25752–61. 10.1074/jbc.270.43.25752 7592757

[11] Ramasamy R Yan SF Schmidt AM. RAGE: therapeutic target and biomarker of the inflammatory response—The evidence mounts. J Leukoc Biol. (2009) 86:505–12. 10.1189/jlb.0409230 19477910

[12] Chitrabamrung S Rubin RL Tan EM. Serum deoxyribonuclease I and clinical activity in systemic lupus erythematosus. Rheumatol Int. (1981) 1:55–60. 10.1007/BF00541153 6287560

[13] Skiljevic D Jeremic I Nikolic M Andrejevic S Sefik-Bukilica M Stojimirovic B. Serum DNase I activity in systemic lupus erythematosus: correlation with immunoserological markers, the disease activity and organ involvement. Clin Chem Lab Med. (2013) 51:1083–91. 10.1515/cclm-2012-0521 23183758

[14] Tsukumo S Yasutomo K. DNaseI in pathogenesis of systemic lupus erythematosus. Clin Immunol. (2004) 113:14–8. 10.1016/j.clim.2004.05.009 15380524

[15] Abdulahad DA Westra J Bijzet J Limburg PC Kallenberg CG Bijl M. High mobility group box 1 (HMGB1) and anti-HMGB1 antibodies and their relation to disease characteristics in systemic lupus erythematosus. Arthritis Res Ther. (2011) 13:R71. 10.1186/ar3332 21548924 PMC3218880

[16] Lu M Yu S Xu W Gao B Xiong S. HMGB1 promotes systemic lupus erythematosus by enhancing macrophage inflammatory response. J Immunol Res. (2015) 2015:946748. 10.1155/2015/946748 26078984 PMC4452473

[17] Cully M. Connective tissue diseases: HMGB1 helps elicit anti-dsDNA antibody production in SLE. Nat Rev Rheumatol. (2013) 9:321. 10.1038/nrrheum.2013.75 23670137

[18] Ramsland PA Farrugia W Bradford TM Sardjono CT Esparon S Trist HM. Structural basis for Fc gammaRIIa recognition of human IgG and formation of inflammatory signaling complexes. J Immunol. (2011) 187:3208–17. 10.4049/jimmunol.1101467 21856937 PMC3282893

[19] Niewold TB. Interferon alpha as a primary pathogenic factor in human lupus. J Interferon Cytokine Res. (2011) 31:887–92. 10.1089/jir.2011.0071 21923413 PMC3234490

[20] Crow MK. Type I interferon in the pathogenesis of lupus. J Immunol. (2014) 192:5459–68. 10.4049/jimmunol.1002795 24907379 PMC4083591

[21] Fitzgerald-Bocarsly P Dai J Singh S. Plasmacytoid dendritic cells and type I IFN: 50 years of convergent history. Cytokine Growth Factor Rev. (2008) 19:3–19. 10.1016/j.cytogfr.2007.10.006 18248767 PMC2277216

[22] Carvalheiro T Rodrigues A Lopes A Ines L Velada I Ribeiro A. Tolerogenic versus inflammatory activity of peripheral blood monocytes and dendritic cells subpopulations in systemic lupus erythematosus. Clin Dev Immunol. (2012) 2012:934161. 10.1155/2012/934161 22969819 PMC3437291

[23] Henriques A Ines L Carvalheiro T Couto M Andrade A Pedreiro S. Functional characterization of peripheral blood dendritic cells and monocytes in systemic lupus erythematosus. Rheumatol Int. (2012) 32:863–9. 10.1007/s00296-010-1709-6 21221593

[24] Hansmann L Groeger S von Wulffen WBein G Hackstein H. Human monocytes represent a competitive source of interferon-alpha in peripheral blood. Clin Immunol. (2008)127:252–64. 10.1016/j.clim.2008.01.014 18342575

[25] Lee PY Weinstein JS Nacionales DC Scumpia PO Li Y Butfiloski E. A novel type I IFN-producing cell subset in murine lupus. J Immunol. (2008) 180:5101–8. 10.4049/jimmunol.180.7.5101 18354236 PMC2909121

[26] Gaynor B Putterman C Valadon P Spatz L Scharff MD Diamond B. Peptide inhibition of glomerular deposition of an anti-DNA antibody. Proc Natl Acad Sci USA. (1997) 94:1955–60. 10.1073/pnas.94.5.1955 9050886 PMC20024

[27] Fragoso-Loyo H Cabiedes J Orozco-Narvaez A Davila-Maldonado L Atisha-Fregoso Y Diamond B. Serum and cerebrospinal fluid autoantibodies in patients with neuropsychiatric lupus erythematosus. Implications for diagnosis and pathogenesis. PLoS ONE (2008) 3:e3347. 10.1371/journal.pone.0003347 18836530 PMC2556096

[28] Hanly JG Robichaud J Fisk JD. Anti-NR2 glutamate receptor antibodies and cognitive function in systemic lupus erythematosus. J Rheumatol. (2006) 33:1553–8. 16881112

[29] Harrison MJ Ravdin LD Lockshin MD. Relationship between serum NR2a antibodies and cognitive dysfunction in systemic lupus erythematosus. Arthritis Rheum. (2006) 54:2515–22. 10.1002/art.22030 16868972

[30] Husebye ES Sthoeger ZM Dayan M Zinger H Elbirt D Levite M. Autoantibodies to a NR2A peptide of the glutamate/NMDA receptor in sera of patients with systemic lupus erythematosus. Ann Rheum Dis. (2005) 64:1210–3. 10.1136/ard.2004.029280 15708887 PMC1755620

[31] Lapteva L Nowak M Yarboro CH Takada K Roebuck-Spencer T Weickert T. Anti-N-methyl-D-aspartate receptor antibodies, cognitive dysfunction, and depression in systemic lupus erythematosus. Arthritis Rheum. (2006) 54:2505–14. 10.1002/art.22031 16868971

[32] Putterman C Diamond B. Immunization with a peptide surrogate for double-stranded DNA (dsDNA) induces autoantibody production and renal immunoglobulin deposition. J Exp Med. (1998) 188:29–38. 10.1084/jem.188.1.29 9653081 PMC2525538

[33] Sharma A Isenberg D Diamond B. Studies of human polyclonal and monoclonal antibodies binding to lupus autoantigens and cross-reactive antigens. Rheumatology (2003) 42:453–63. 10.1093/rheumatology/keg161 12626796

[34] Wang H Bloom O Zhang M Vishnubhakat JM Ombrellino M Che J. HMG-1 as a late mediator of endotoxin lethality in mice. Science (1999) 285:248–51. 10.1126/science.285.5425.248 10398600

[35] Yang H Hreggvidsdottir HS Palmblad K Wang H Ochani M Li J. A critical cysteine is required for HMGB1 binding to toll-like receptor 4 and activation of macrophage cytokine release. Proc Natl Acad Sci USA. (2010) 107:11942–7. 10.1073/pnas.1003893107 20547845 PMC2900689

[36] Ostreiko KK Tumanova IA Sykulev Yu K. Production and characterization of heat-aggregated IgG complexes with pre-determined molecular masses: light-scattering study. Immunol Lett. (1987) 15:311–6. 10.1016/0165-2478(87)90134-9 3692537

[37] Obermoser G Pascual V. The interferon-alpha signature of systemic lupus erythematosus. Lupus (2010) 19:1012–9. 10.1177/0961203310371161 20693194 PMC3658279

[38] Lovgren T Eloranta ML Kastner B Wahren-Herlenius M Alm GV Ronnblom L. Induction of interferon-alpha by immune complexes or liposomes containing systemic lupus erythematosus autoantigen- and Sjogren's syndrome autoantigen-associated RNA. Arthritis Rheum. (2006) 54:1917–27. 10.1002/art.21893 16729300

[39] Urbonaviciute V Furnrohr BG Meister S Munoz L Heyder P De Marchis F. Induction of inflammatory and immune responses by HMGB1-nucleosome complexes: implications for the pathogenesis of SLE. J Exp Med. (2008) 205:3007–18. 10.1084/jem.20081165 19064698 PMC2605236

[40] Wen Z Xu L Chen X Xu W Yin Z Gao X. Autoantibody induction by DNA-containing immune complexes requires HMGB1 with the TLR2/microRNA-155 pathway. J Immunol. (2013) 190:5411–22. 10.4049/jimmunol.1203301 23616573

[41] Banchereau J Pascual V. Type I interferon in systemic lupus erythematosus and other autoimmune diseases. Immunity (2006) 25:383–92. 10.1016/j.immuni.2006.08.010 16979570

[42] Oon S Wilson NJ Wicks I. Targeted therapeutics in SLE: emerging strategies to modulate the interferon pathway. Clin Transl Immunol. 5:e79. 10.1038/cti.2016.26 27350879 PMC4910120

[43] Wu YW Tang W Zuo JP. Toll-like receptors: potential targets for lupus treatment. Acta Pharmacol Sin. (2015) 36:1395–407. 10.1038/aps.2015.91 26592511 PMC4816237

[44] Guiducci C Gong M Xu Z Gill M Chaussabel D Meeker T. TLR recognition of self nucleic acids hampers glucocorticoid activity in lupus. Nature (2010) 465:937–41. 10.1038/nature09102 20559388 PMC2964153

[45] Kiefer K Oropallo MA Cancro MP Marshak-Rothstein A. Role of type I interferons in the activation of autoreactive B cells. Immunol Cell Biol. (2012) 90:498–504. 10.1038/icb.2012.10 22430248 PMC3701256

[46] Tough DF. Modulation of T-cell function by type I interferon. Immunol Cell Biol. (2012) 90:492–7. 10.1038/icb.2012.7 22391814

[47] Wu X Peng SL. Toll-like receptor 9 signaling protects against murine lupus. Arthritis Rheum. (2006) 54:336–42. 10.1002/art.21553 16385525

[48] van Holten J Reedquist K Sattonet-Roche P Smeets TJ Plater-Zyberk C Vervoordeldonk MJ Treatment with recombinant interferon-beta reduces inflammation and slows cartilage destruction in the collagen-induced arthritis model of rheumatoid arthritis. Arthritis Res Ther. (2004) 6:R239–49. 10.1186/ar1165 15142270 PMC416442

[49] van Holten J Plater-Zyberk C Tak PP Interferon-beta for treatment of rheumatoid arthritis? Arthritis Res. (2002) 4:346–52. 10.1186/ar598 12453310 PMC153843

[50] Trinchieri G Type I interferon: friend or foe? J Exp Med. (2010) 207:2053–63. 10.1084/jem.20101664 20837696 PMC2947062

[51] Yarilina A DiCarlo E Ivashkiv LB. Suppression of the effector phase of inflammatory arthritis by double-stranded RNA is mediated by type I IFNs. J Immunol. (2007) 178:2204–11. 10.4049/jimmunol.178.4.2204 17277125

[52] Bertheloot D Naumovski AL Langhoff P Horvath GL Jin T Xiao TS. RAGE enhances TLR responses through binding and internalization of RNA. J Immunol. (2016) 197:4118–26. 10.4049/jimmunol.1502169 27798148 PMC6062438

[53] Sirois CM Jin T Miller AL Bertheloot D Nakamura H Horvath GL. RAGE is a nucleic acid receptor that promotes inflammatory responses to DNA. J Exp Med. (2013) 210:2447–63. 10.1084/jem.20120201 24081950 PMC3804942

[54] Xu J Jiang Y Wang J Shi X Liu Q Liu Z. Macrophage endocytosis of high-mobility group box 1 triggers pyroptosis. Cell Death Differ. (2014) 21:1229–39. 10.1038/cdd.2014.40 24769733 PMC4085529

[55] Yanai H Chiba S Ban T Nakaima Y Onoe T Honda K. Suppression of immune responses by nonimmunogenic oligodeoxynucleotides with high affinity for high-mobility group box proteins (HMGBs). Proc Natl Acad Sci USA. (2011) 108:11542–7. 10.1073/pnas.1108535108 21709231 PMC3136288

[56] Watanabe H Watanabe KS Hiramatsu S Zeggar S Katsuyama E Tatebe N Anti-high mobility group Box1 amliorates albuminuria in MRL/lpr lupus prone mice. Mol Ther Methods Clin Dev. (2017) 6:31–9. 10.1016/j.omtm.2017.05.006 28649578 PMC5472134

[57] Zhang C Li C Jia S Yao P Yang Q Zhang Y. High-mobility group box 1 inhibition alleviates lupus-like disease in BXSB mice. Scand J Immunol. (2014) 79:333–7. 10.1111/sji.12165 24612327

