# Supplementary figures and images for: DNA-Mediated Interferon Signature Induction by SLE Serum Occurs in Monocytes Through Two Pathways: A Mechanism to Inhibit Both Pathways
Source: Front Immunol. 2018 Dec 11;9:2824. doi: 10.3389/fimmu.2018.02824 (PMC6297782; doi:10.3389/fimmu.2018.02824)

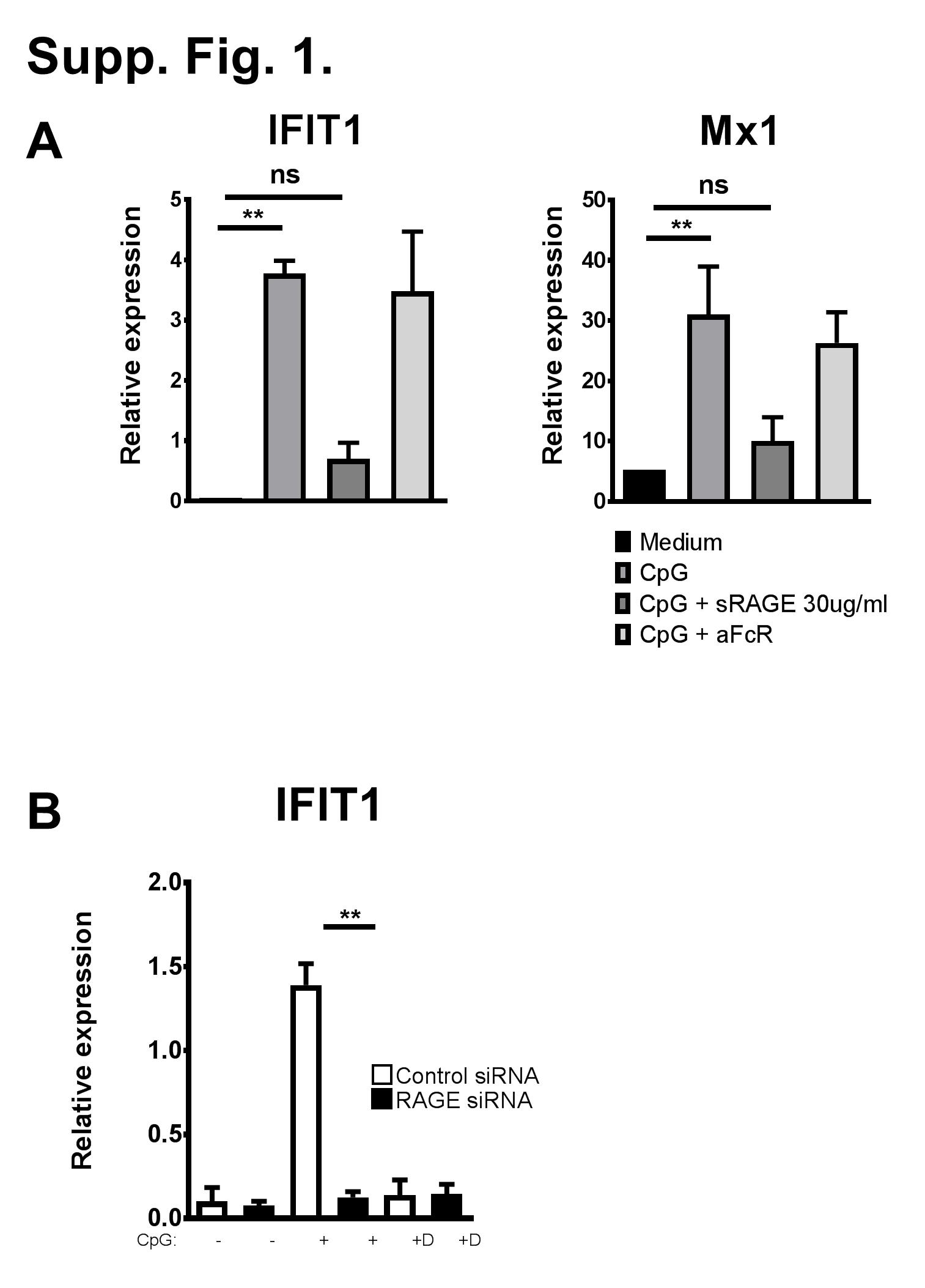

Supplement: Supplementary Figure 1 — (A,B) ISG induction by CpG is inhibited by blocking RAGE, but not FcRIIa. (A) Primary human monocytes were incubated with CpG alone or with soluble RAGE (sRAGE) or with blocking anti-FcRIIa antibody for 4 hours. ISG levels were assessed by qPCR. (B) Primary human monocytes were transfected with control or RAGE siRNA as noted in the methods section. Cells were then treated with CpG alone (+) or with DWEYS (+D) for 4 h. IFIT1 levels were assessed by qPCR. Results indicate mean ± SD of three independent experiments. *P < 0.05; **P < 0.01; ***P < 0.001. [file Image_1.JPEG]

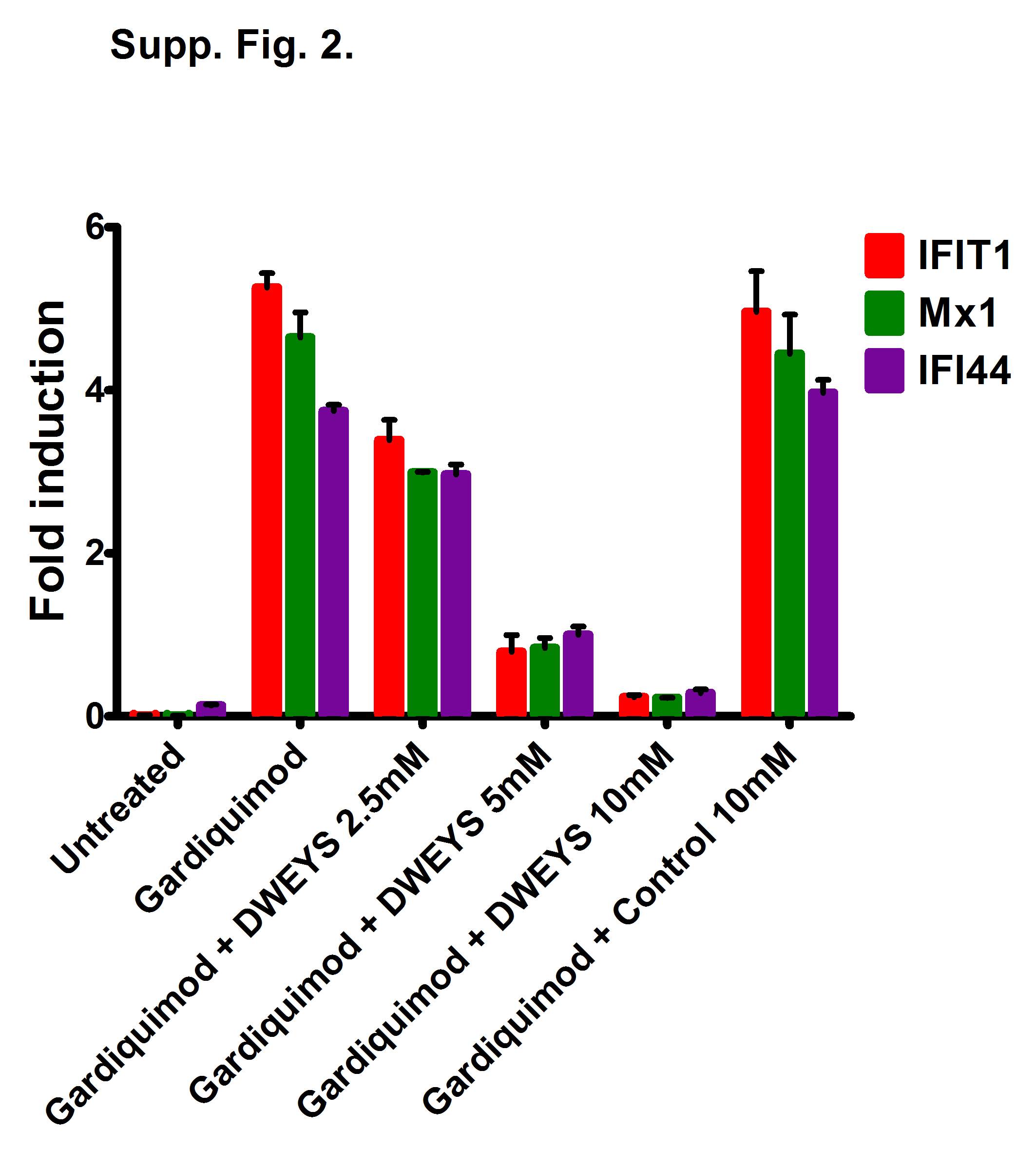

Supplement: Supplementary Figure 2 — DWEYS inhibited ISG induction by TLR7 agonist, gardiquimod. Primary human monocytes were incubated with gardiquimod alone, or with soluble DWEYS peptide at indicated molar excess for 4 h. ISG levels were assessed by qPCR as in all other experiments. [file Image_2.JPEG]
